# Assessment of Strategies and Epidemiological Characteristics of Tuberculosis in Henan Province, China: Observational Study

**DOI:** 10.2196/24830

**Published:** 2021-01-22

**Authors:** Hui Jiang, Guolong Zhang, Jinfeng Yin, Dongyang Zhao, Fangchao Liu, Yuxia Yao, Chao Cai, Jiying Xu, Xinwei Li, Wangli Xu, Weimin Li

**Affiliations:** 1 Beijing Chest Hospital Capital Medical University Beijing China; 2 Beijing Tuberculosis and Thoracic Tumor Research Institute Beijing China; 3 Institute of Tuberculosis Control and Prevention Henan Center for Disease Control and Prevention Henan China; 4 Beijing Youan Hospital Capital Medical University Beijing China; 5 School of Statistics Renmin University of China Beijing China; 6 Beijing Municipal Key Laboratory of Clinical Epidemiology School of Public Health Capital Medical University Beijing China; 7 National Tuberculosis Clinical Lab of China Beijing Tuberculosis and Thoracic Tumour Research Institute Beijing Key Laboratory in Drug Resistance Tuberculosis Research Beijing China

**Keywords:** notified pulmonary tuberculosis, Tuberculosis Information Management System, epidemiological characteristics, dynamic compartmental model, policy evaluation

## Abstract

**Background:**

In 2005, China established an internet-based Tuberculosis Information Management System (TBIMS) to monitor changes in tuberculosis (TB). Many scholars have conducted epidemiological research using TBIMS; however, few studies assessing control strategies have been performed based on this platform data. Henan province is a high TB incidence area in China where, in addition to following the nationwide TB strategies, a series of local intervention combinations have been implemented.

**Objective:**

Our study aims to evaluate the impact of nationwide TB intervention combinations on epidemiological changes and determine whether Henan province can achieve the World Health Organization’s (WHO) goal of reducing TB incidence by 50% and TB mortality by 75% by the year 2025.

**Methods:**

We used descriptive statistical methods to show the spatial and temporal distribution of pulmonary tuberculosis (PTB) reported to the TBIMS database from 2005 to 2018, and logistic regression analysis was performed to identify the risk factors of bacteriological-positive TB. The dynamic compartmental model and Bayesian melding approach was adopted to estimate the burden of TB under the impact of different TB control policies.

**Results:**

In total, 976,526 PTB cases were notified to the TBIMS in Henan in a period of 14 years. Although the overall incidence of PTB declined from 91.4/10^5^ to 58.5/10^5^, and the overall incidence of bacteriological-positive PTB declined from 44.5/10^5^ to 14.7/10^5^, the WHO’s 2025 goal could not be met. The distribution of high incidence and poverty-stricken counties were basically overlapped. Men, farmers and herdsmen (in rural areas), and subjects aged ≥60 years were more likely to develop bacteriological-positive PTB. The increasing treatment success for drug-susceptible tuberculosis and multidrug-resistant tuberculosis has not provided the desired reduction in incidence and mortality.

**Conclusions:**

To achieve the targeted goal, while improving the cure rate of TB, new active (rather than passive) detection and intervention strategies should be formulated based on epidemiological characteristics in Henan province.

## Introduction

Implementation of the directly observed treatment short-course (DOTS) chemotherapy strategy led to a 65% reduction in the prevalence of smear-positive tuberculosis (TB) in China between 1990 and 2010 [1]. Nevertheless, China still had the second-highest number of TB infections globally [2]. In 2005, China established an internet-based Tuberculosis Information Management System (TBIMS) as the national TB surveillance system, and all Chinese TB health facilities have been required to report diagnosed pulmonary tuberculosis (PTB) cases directly into the TBIMS [3]. The TBIMS platform allows for real-time monitoring of TB diagnosis, treatment, and outcomes in China, especially for PTB. Recently, many scholars have conducted epidemiological investigations using TBIMS; however, few studies have assessed control strategies based on this platform data.

The World Health Organization’s (WHO) 2025 goal was to reduce TB incidence by 50% and TB mortality by 75% [4] from 2015 to 2025. Recently, research studies have shown that China is unlikely to meet the global TB-related targets by intensifying its current strategies, which were passive measures taken only for TB symptomatic persons [5,6]. Henan province is a high incidence area, accounting for 10% of TB infections in China. In addition to following the nationwide TB strategies, Henan province has also implemented a series of nonfragmented local intervention combinations, including an annual investment from the local authorities of 1.42 million US dollars to purchase antituberculosis drugs and diagnostic reagents since 2010, the provision of free screening for latent TB infection to enrolled first-year students since 2017, and a payment plan targeting single-disease TB treatment since 2018. However, whether these measures would change the epidemiological characteristics and help Henan province achieve the WHO’s goal for 2025 has not been determined.

In this study, based on the TBIMS database, we collected details of PTB as well as demographic, epidemiological, geographical, and laboratory information and related policies in Henan province for 2005-2018. We aim to explore whether the epidemiological characteristics of TB have changed in the past 14 years under the guidance of the national and provincial policies, and to use a dynamic compartmental model to evaluate various TB control and prevention policies in order to determine whether Henan province can achieve the WHO’s 2025 goal.

## Methods

### Data Collection

Since January 1, 2005, PTB cases are reported to the TBIMS, the national TB surveillance system, within 24 hours of diagnosis [3]. From the TBIMS database, we collected information from PTB cases on basic demographic information (sex, birth date, home address, occupation, and treatment of classification), time of illness onset and diagnosis, and laboratory outcomes (sputum smear results and sputum culture results). The percentage values of multidrug-resistant tuberculosis (MDR-TB) infections in all cases, new patients, and re-treated patients were obtained by analysis of multidrug resistance of tuberculosis. The mortality was obtained from the national disease surveillance system’s tuberculosis death analysis report and the Ministry of Health’s prevalence survey in 2010.

### Data Analysis

Descriptive statistical methods were adopted to analyze continuous variables and categorical variables. The annual incidence rates of PTB (per 100,000 people) and bacteriological-positive PTB were calculated. We used the Arc Map’s (version 10.2; ESRI Inc) ring map to show the spatiotemporal patterns of PTB incidence. In order to illustrate the seasonal patterns of PTB in different regions, we created heatmaps for the proportion of PTB cases identified during each month of the year. A hierarchical clustering method was used to identify similar regions based on the overall and bacteriological-positive PTB incidence rates, which were compared with the distribution of poverty-stricken regions in Henan province. Univariable and multivariable logistic regression models were applied in order to investigate the factors associated with bacteriological-positive PTB, and unadjusted odds ratios (OR) and adjusted odds ratios (AOR) were also estimated.

We used a dynamic compartmental model [5,7] to predict the incidence and mortality of TB epidemics, and the main parameters included natural history parameters, mortality rate or birth rate, and program parameters such as patient visit rate and long-term cure rate. We found the present background parameters values through a review of the literature [5,6], including the long-term cure rate for new cases and re-treatment cases in different medical settings. Based on experts' opinions and a local epidemiological survey from Henan Center for Disease Control and Prevention, we selected and set the local background parameters and further simulated 4 main scenarios for the future. We assumed that these scenarios would be implemented in 2018 and estimated their effect by 2025 using the model. In scenario 1 (the current status in Henan), we estimated the ranges of the decline in incidence and mortality if the current strategies are maintained until 2025. In scenario 2, we estimated the change of incidence and mortality by year if the treatment success rate for drug-susceptible tuberculosis (DS-TB) increased to 92% for new treatment and 90% for re-treatment. In scenario 3, we considered using better diagnosis technologies and increasing treatment success for MDR-TB to show the impact of the measures, and the long-term cure rate with a second-line drug for MDR-TB was 82%. In scenario 4, a combination of all conducted measures was represented; that is, scenarios 1, 2, and 3 were delivered simultaneously. A detailed description of the statistical analyses is provided in Multimedia Appendix 1.

In addition, the data of TB burden in Henan province were used to calibrate the model by adopting the Bayesian melding approach [8]. Then, the impact of intervention strategies on the epidemiology of TB was predicted using the fitted model. We predicted the incidence, mortality, and multidrug-resistant percentage for all cases and new cases for each scenario. The posterior simulations produced 95% credible intervals. Furthermore, the post-2015 global tuberculosis targets were compared with the variation in incidence and mortality that we calculated.

### Ethics Statement

The ethics review committee of the Henan Tuberculosis Control Institute provided approval for this study. Additionally, patients’ information was anonymized to ensure privacy.

## Results

### Demographic Characteristics

**Figure 1:**
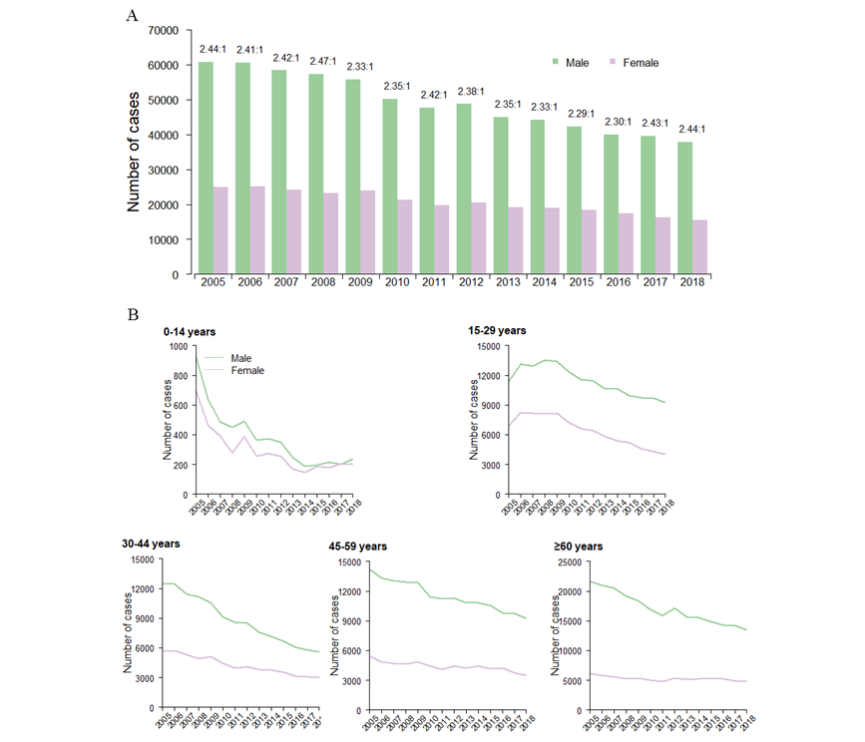
Number of pulmonary tuberculosis (PTB) cases by age and sex in Henan province, China, from 2005 to 2018. A: Number of PTB cases by sex and year. B: Number of PTB cases by age group, sex, and year.

A total of 976,526 PTB cases were reported to TBIMS in the 2005–2018 period. Of these, 381,598 (39.08%) were cases of bacteriological-positive tuberculosis. In all PTB cases, the overall male-to-female ratio was 2.38:1; however, this pattern was not uniform across the years (Figure 1A), and the gender difference in the 0-14 years age group was the lowest (Figure 1B). The median age was 48 (IQR 28–63) years, and the median age for bacteriological-positive cases (51 years, IQR 31-65) was higher than for negative cases (47 years, IQR 28-63).

Moreover, 95.0% of patients were new cases, and 98.2% had received antituberculosis therapy (Table 1).

**Table 1:** Characteristics of pulmonary tuberculosis (PTB) cases in Henan province, China, from 2005 to 2018.

Characteristic		Total PTB cases (n=976,526)	Laboratory results	
			Bacteriological-positive (n=381,598)	Bacteriological-negative (n=526,888)
**Gender**				
	Male, n (%)	687,996 (70.45)	287,143 (74.54)	358,916 (68.12)
	Female, n (%)	288,530 (29.55)	98,055 (25.46)	167,972 (31.88)
**Age groups in years, n (%)**				
	0-14	9,434 (0.97)	1,086 (0.28)	4,668 (0.89)
	15-29	248,376 (25.43)	87,272 (22.66)	137,771 (26.15)
	30-44	182,613 (18.70)	69,732 (18.10)	99,220 (18.83)
	45-59	223,073 (22.84)	90,222 (23.42)	120,923 (22.95)
	≥60	312,620 (32.01)	136,734 (35.50)	164,116 (31.15)
	Unknown	410 (0.04)	152 (0.04)	190 (0.04)
**Occupation, n (%)**				
	Nursery children	307 (0.03)	8 (0.00)	79 (0.01)
	Scattered children	729 (0.07)	36 (0.01)	246 (0.05)
	Students	47,760 (4.89)	11,171 (2.90)	29,690 (5.63)
	Workers	26,229 (2.69)	9,924 (2.58)	14,135 (2.68)
	Farmers and herdsmen	795,893 (81.50)	325,259 (84.44)	425,466 (80.75)
	Commercial service stratum	4704 (0.48)	1607 (0.42)	2650 (0.50)
	Others	98,414 (10.08)	36,181 (9.39)	53,395 (10.13)
	Unknown	2490 (0.25)	1012 (0.26)	1227 (0.23)
**Residence, n (%)**				
	Rural	786,210 (80.51)	317,547 (82.44)	419,554 (79.63)
	Urban	180,375 (18.47)	63,582 (16.51)	102,130 (19.38)
	Unknown	9941 (1.02)	4069 (1.06)	5204 (0.99)
**Treatment of classification, n (%)**				
	New case	927,250 (94.95)	358,170 (92.98)	505,423 (95.93)
	Re-treated case	49,276 (5.05)	27,028 (7.02)	21,465 (4.07)
**Anti-tuberculosis therapy**				
	Yes	958,965 (98.20)	379,016 (98.44)	517,660 (98.31)
	No	16,978 (1.74)	6017 (1.56)	8917 (1.69)
	Unknown	583 (0.06)	165 (0.04)	311 (0.06)
**Time from illness onset to first hospital visit, in days, n (%)**				
	0-30	646,116 (66.16)	235,774 (61.21)	365,316 (69.33)
	31-60	119,264 (12.21)	50,124 (13.01)	62,298 (11.82)
	61-90	41,171 (4.22)	19,310 (5.01)	19,938 (3.78)
	>90	53,414 (5.47)	26,919 (6.99)	24,511 (4.65)
	Unknown	116,561 (11.94)	53,071 (13.78)	54,825 (10.41)
**Time from onset to confirmation, in days, n (%)**				
	0-30	564,081 (57.76)	205,505 (53.35)	318,226 (60.40)
	31-60	192,248 (19.69)	76,306 (19.81)	103,770 (19.69)
	61-90	68,368 (7.00)	30,570 (7.94)	34,263 (6.50)
	>90	96,450 (9.88)	48,874 (12.69)	44,032 (8.36)
	Unknown	55,379 (5.67)	23,943 (6.22)	26,597 (5.05)
**Time from onset to treatment end, in months, n (%)**				
	0-6	47,621 (4.88)	19,284 (5.01)	24,999 (4.74)
	6-12	715,251 (73.24)	277,827 (72.13)	404,604 (76.79)
	>12	60,994 (6.25)	22,312 (5.79)	22,390 (4.25)
	Unknown	152,660 (15.63)	65,775 (17.08)	74,895 (14.21)

### Incidence and Seasonality

**Figure 2:**
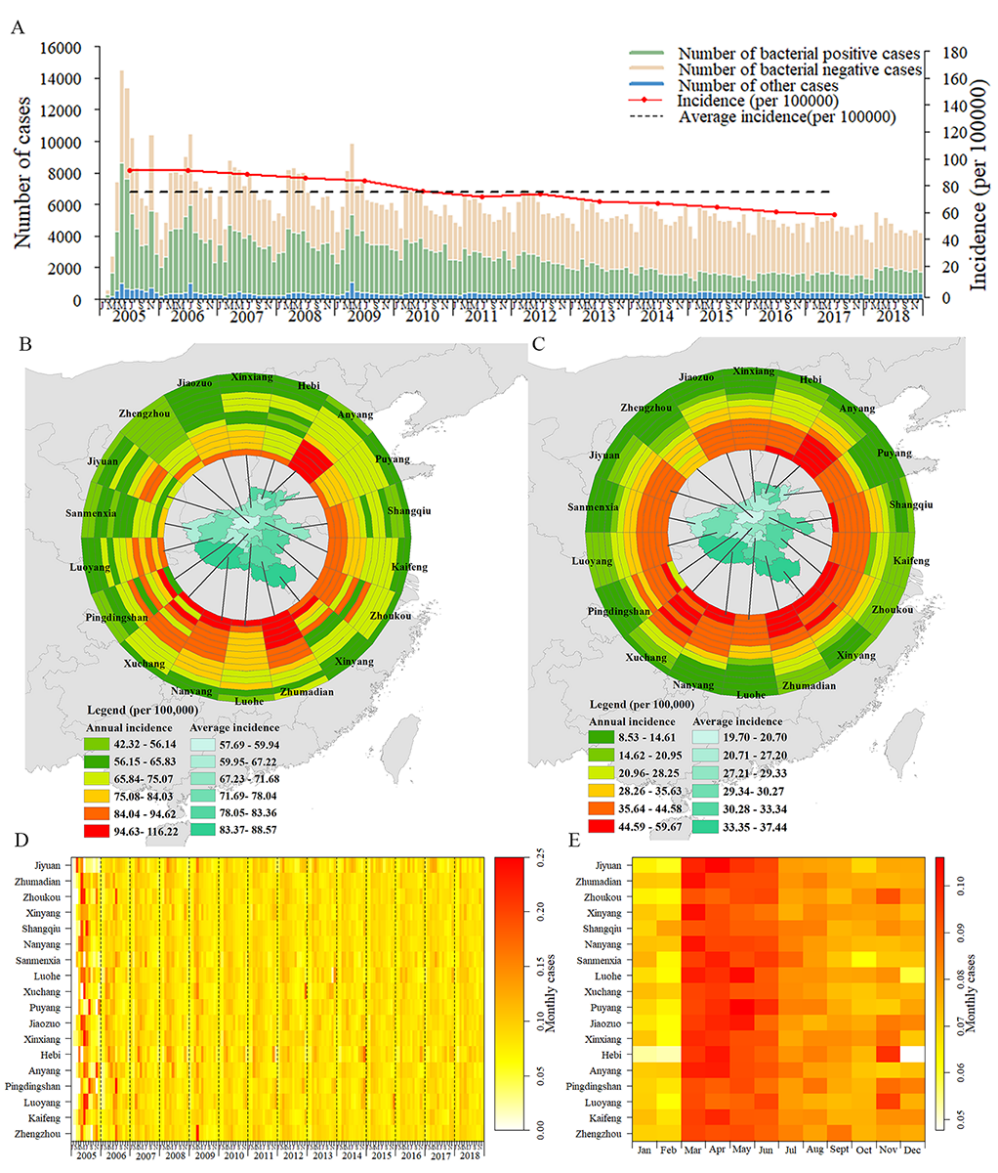
Spatiotemporal distribution of pulmonary tuberculosis (PTB) cases by city and epidemic curve in Henan province, China, from 2005 to 2018. A: PTB epidemic curve on the number of cases reported weekly. B: Spatiotemporal distribution of all PTB cases. C: Spatiotemporal distribution of bacteriological-positive PTB cases. D: Time series for weekly reported PTB cases (standardized by the number of annual cases). E: Cluster analysis for seasonal distribution of PTB cases.

The annual average PTB incidence was 75.3/10^5^, and the overall incidence trend declined from 91.4/10^5^ in 2005 to 58.5/10^5^ in 2017. The annual average incidence for patients with bacteriological-positive PTB was 30.1/10^5^, and it decreased from 44.5/10^5^ in 2005 to 14.7/10^5^ in 2017 (Figure 2A).

PTB incidence showed broad age-specific variations, and patients aged ≥60 years and 15-29 years ranked first and second, respectively. In addition, the incidence in rural regions was higher than in urban areas (*P*<.05; Table 2).

**Figure 3:**
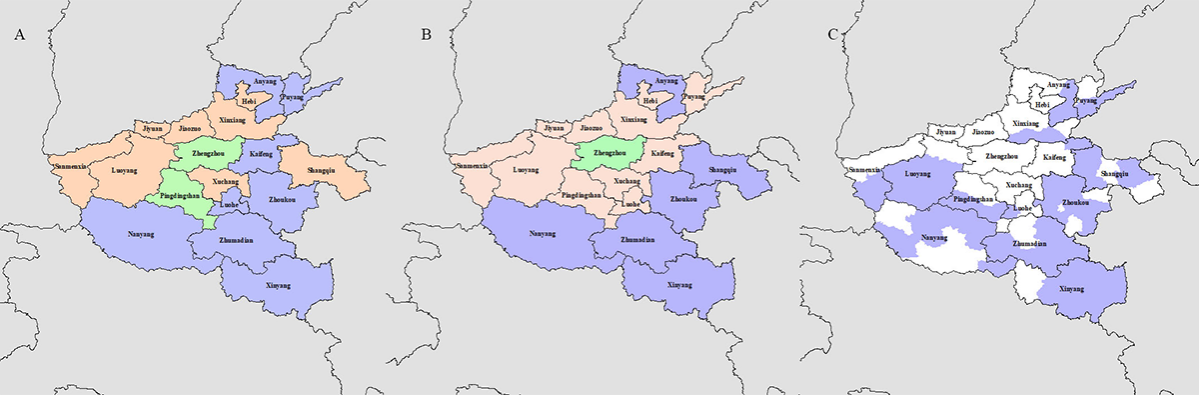
Classification of pulmonary tuberculosis (PTB) epidemiological regions through cluster analysis in Henan province, China, from 2005 to 2018. A: Classification of all PTB epidemiological regions. B: Classification of bacteriological-positive PTB epidemiological regions. C: Distribution of poverty-stricken counties.

As can be observed from the PTB’s geographical distribution across Henan in the 2005-2018 period, the high incidence areas of PTB in Henan province remained unchanged (Figure 2B). In addition, from 2005 to 2010, the overall incidence of bacteriological-positive cases remained high, except in Zhengzhou city (Figure 2C). The incidence rates of PTB, cases of bacteriological-positive tuberculosis, and poverty-stricken counties were basically the same, especially in Nanyang city, Xinyang city, Zhoukou city, and Zhumadian city (Figure 3A-C).

**Table 2:** Incidence of pulmonary tuberculosis (PTB) cases by age group and residence in Henan province, China, from 2005 to 2017.

Characteristic			Year (2005-2017)																						
			2005		2006	2007		2008		2009		2010		2011		2012		2013		2014		2015		2016	2017
**Residence**																									
	Urban	46.4		48.1		42.9	39.6		40.8		35.1		31.0		31.7		29.8		29.8		27.1		25.6		25.8
	Rural	108.5		110.6		110.4	109.6		108.2		100.5		98.1		103.1		96.9		96.9		95.7		92.3		90.7
**Age group, in years**																									
	0-14	8.1		5.63		4.6	3.8		4.8		3.1		3.3		3.2		2.1		1.6		1.9		1.9		2.0
	15-29	—^a^		104.2		100.9	103.9		103.5		84.5		82.9		83.2		81.7		77.4		74.9		71.9		74.6
	30-44	—		72.2		68.5	68.8		69.1		62.5		58.4		61.9		59.1		54.8		53.7		48.6		48.7
	45-59	—		103.7		96.0	86.6		87.1		90.8		86.8		82.7		73.3		76.0		71.5		67.2		60.1
	≥60	—		237.5		243.0	218.2		186.0		182.0		156.2		158.2		143.6		147.7		135.1		127.3		121.8

^a^ — not available.

On the provincial scale, it can be observed that people developed PTB throughout the year, while at the city-level, the incidence did not reach a peak at the same time across Henan; nevertheless, the annual seasonal peaks were noticeable. A seasonal pattern was observed in March, April, May, and June, and the peak season occurred in March and April. Cases decreased during early June and after mid-October, and the PTB cases increased again, with transmission reaching a small peak, in November. The 2 months with the lowest PTB incidence were January and February (Figure 2D-E).

### Changing Trends of Bacteriological-Positive Results

**Figure 4:**
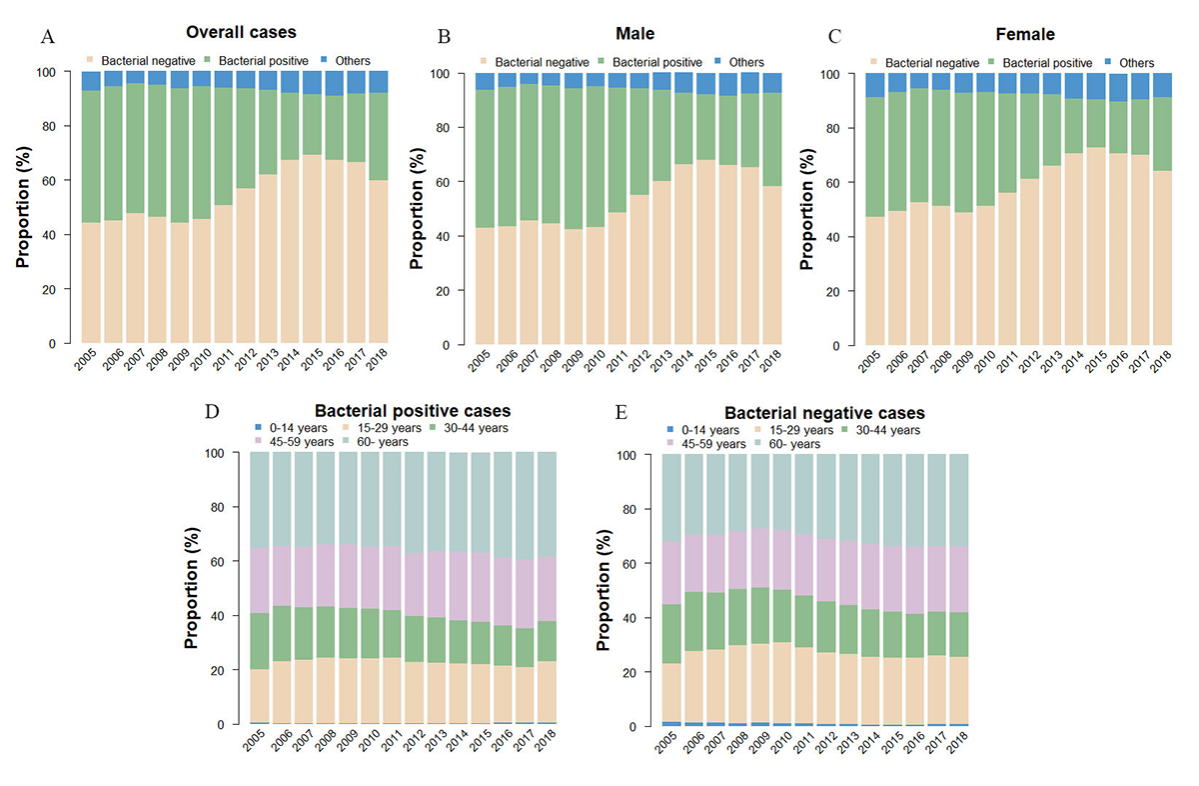
Proportion of laboratory-tested pulmonary tuberculosis (PTB) cases by sex and bacteriological type in Henan province, China, from 2005 to 2018. A: based on overall cases; B: based on male cases; C: based on female cases; D: based on bacteriological-positive cases; E: based on bacteriological-negative cases.

Bacteriological results showed that the proportion of bacteriological-positive cases decreased from 48.79% in 2005 to 23.64% in 2016. However, the proportion of bacteriological-positive infection rose in 2017 and 2018 to 25.20% and 32.27%, respectively (Figure 4A). Further, the proportion of males with bacteriological-positive tuberculosis was higher than that of females (Figure 4B-C). In addition, the proportion of patients with bacteriological-positive infection in the ≥60 years age group was the highest, and the proportion increased continuously from 35.42% in 2005 to 38.42% in 2018 (Figure 4D). The proportion of patients with bacteriological-negative infection across different age groups was consistent with that of patients with bacteriological-positive infection (Figure 4E).

### Risk Factors for Bacteriological-Positive Infection

Male sex, residence in rural areas, and re-treated cases were factors associated with the development of bacteriological-positive PTB, and the AORs for these groups were 1.32, 1.17, and 1.70, respectively. In addition, a trend of substantial increase in PTB risk with bacteriological-positive infection was observed with increasing age; the AORs were 1.91, 1.92, 1.97, and 2.17 for the 15-29, 30-44, 45-59, and ≥60 years age groups, respectively. Moreover, workers, farmers and herders, and the commercial service stratum were more susceptible to developing bacteriological-positive PTB than nursery children and students; the AORs for these groups were 3.78 (95% CI 1.93-8.54), 3.69 (95% CI 1.88-8.34), and 3.44 (95% CI 1.75-7.79), respectively (Table 3).

**Table 3:** Risk factors for bacteriological-positive infection cases in Henan province, 2005-2018.

Characteristic		Laboratory results, n (%)			Odds ratio (95% CI)		Adjusted odds ratio (95% CI)
			Bacteriological-positive	Bacteriological-negative			
**Sex**							
	Male		287,143 (74.54)	358,916 (68.12)		1.37 (1.35-1.38)	1.32 (1.31-1.34)
	Female		98,055 (25.46)	167,972 (31.88)		1	1
**Age groups, in years**							
	0-14		1086 (0.28)	4668 (0.89)		1	1
	15-29		87,272 (22.67)	137,771 (26.16)		2.72 (2.54-2.91)	1.91 (1.79-2.06)
	30-44		69,732 (18.11)	99,220 (18.84)		3.02 (2.82-3.23)	1.92 (1.79-2.06)
	45-59		90,222 (23.43)	120,923 (22.96)		3.20 (3.00-3.42)	1.97 (1.84-2.12)
	≥60		136,734 (35.51)	164,116 (31.16)		3.58 (3.35-3.82)	2.17 (2.02-2.33)
**Occupation**							
	Nursery children		8 (0.00)	79 (0.02)		1	1
	Scattered children		36 (0.01)	246 (0.06)		1.44 (0.67-3.46)	1.33 (0.61-3.22)
	Students		11,171 (2.91)	29,690 (5.65)		3.71 (1.91-8.35)	2.22 (1.14-5.02)
	Workers		9924 (2.58)	14,135 (2.69)		6.93 (3.56-15.58)	3.78 (1.93-8.54)
	Farmers and herdsmen		325,259 (84.66)	425,466 (80.94)		7.54 (3.88-16.96)	3.69 (1.88-8.34)
	Commercial service stratum		1607 (0.42)	2650 (0.50)		5.98 (3.07-13.48)	3.44 (1.75-7.79)
	Others		36,181 (9.42)	53,395 (10.16)		6.69 (3.44-15.04)	3.61 (1.84-8.15)
**Residence**							
	Urban		63,582 (16.68)	102,130 (19.58)		1	1
	Rural		317,547 (83.32)	419,554 (80.42)		1.21 (1.20-1.22)	1.17 (1.15-1.18)
**Treatment history**							
	New case		358,170 (92.98)	505,423 (95.93)		1	1
	Retreated case		27,028 (7.02)	21,465 (4.07)		1.77 (1.74-1.80)	1.70 (1.66-1.73)

### Impact of Interventions on TB Control Policies

**Figure 5:**
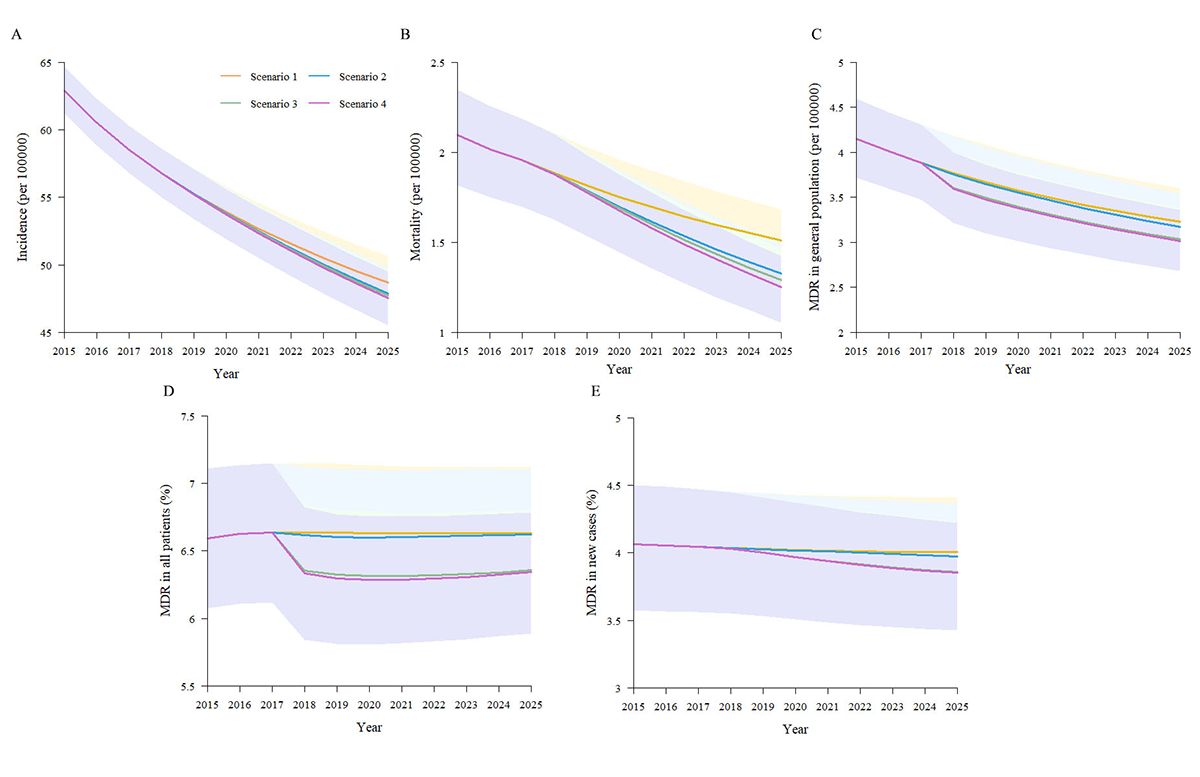
Expected impact of different scenarios of tuberculosis (TB) control on the prevalence of TB in Henan province, China, 2015-2025. A: Expected impact of different scenarios of TB control on the annual TB incidence. B: Expected impact of different scenarios of TB control on the annual TB mortality. C: Expected impact of different scenarios of TB control on the percentage of multidrug-resistant tuberculosis (MDR-TB) in the general population. D: Expected impact of different scenarios of TB control on the percentage of MDR-TB in all TB cases. E: Expected impact of different scenarios of TB control on the percentage of MDR-TB in new TB cases.

In scenario 1 (the current status in Henan), if the strategies are kept unchanged, the incidence and mortality are expected to gradually decline by 22.6% (95% CI 21.7%-23.6%) and 27.9% (95% CI 27.0%-28.3%), respectively, from 2015 to 2025 (Figure 5A-B, Table 4).

**Table 4:** Impacts of different intervention scenarios on pulmonary tuberculosis (PTB) epidemiology in Henan province, China, from 2015 to 2025.

Scenario	Tuberculosis incidence		Tuberculosis-related death	
	Cumulative number, n (95% CI)	Cumulative reduction, % (95% CI)	Cumulative number, n (95% CI)	Cumulative reduction, % (95% CI)
1	665,394 (643,266-688,305)	22.61 (21.72-23.62)	21,599 (18,716-24,151)	27.92 (27.02-28.28)
2	662,400 (640,116-685,372)	23.90 (22.96-25.03)	20,741 (17,881-23,273)	36.80 (36.06-38.04)
3	661,772 (639,449-684,757)	24.16 (23.20-25.31)	20,582 (17,725-23,112)	38.47 (37.71-39.85)
4	660,979 (638,633-683,974)	24.46 (23.48-25.63)	20,384 (17,528-22,907)	40.35 (39.39-42.08)

In scenario 2, if the treatment success rate of new and re-treatment cases is increased to 92% and 90%, respectively, the incidence and mortality will be decreased by 23.9% (95% CI 23.0%-25.0%) and 36.8% (95% CI 36.1%-38.0%), respectively, by 2025 (Figure 5A-B, Table 4).

In scenario 3, if 90% of TB smear-positive patients are tested for resistance testing, and the long-term cure rate for MDR-TB can reach 82%, the incidence and mortality was found to decline by 24.2% (95% CI 23.2%-25.3%) and 38.5% (95% CI 37.7%-39.9%), respectively (Figure 5A-B, Table 4). In addition, we observed that the proportions of MDR-TB in all patients with TB and new cases were declining slowly in Henan with the current measures. Moreover, the use of better diagnostic technologies and increasing treatment success for MDR-TB would yield the greatest percentage reduction in MDR-TB in the general population, all patients with TB, and new cases, by 26.9%, 3.6%, and 5.1%, respectively, from 2015 to 2025 (Figure 5C-E).

The greatest reduction was observed in scenario 4, where the combined strategy would yield reductions in incidence and mortality of 24.5% (95% CI 23.5%-25.6%) and 40.4% (95% CI 39.4%-42.1%), respectively, by 2025 (Figure 5A-B, Table 4).

## Discussion

We used a provincial website tuberculosis surveillance dataset spanning 14 years to collect 976,526 PTB cases and 381,598 bacteriological-positive PTB cases beginning from 2005.

Consistent with the trend of substantially decreasing worldwide PTB incidence [9], Henan also witnessed a sharp decline in PTB and bacteriological-positive PTB incidence rates, from 91.4/10^5^ to 58.5/10^5^ and 44.5/10^5^ to 14.7/10^5^, respectively. This result reflects the effectiveness of targeted TB control measures, such as the investment of 1.42 million US dollars to improve the cure rates of DS-TB. It is important to note that the successful treatment of a TB case translates into a reduction in infection sources, which, in turn, reduces transmission and incidence. The post-2015 global TB-related WHO targets propose a reduction of 50.0% in incidence and 75.0% in mortality by 2025 [10]. Recent studies show that, even under the most optimistic circumstances, China is unlikely to meet these global TB-related targets by intensifying its current strategies [5,6]. Our model also shows that although Henan province’s decrease ranges for incidence (22.6%) and mortality (27.9%) are higher than those for China (20.1% and 23.1%, respectively), and even though all the changes and interventions considered in our analysis were implemented, Henan province will also not be able to achieve the post-2025 WHO global target. To reach this target, Henan province should actively detect and treat TB instead of adopting passive strategies [4,6].

The results indicate that in the context of TB prevention and control, more attention should be paid to males, elderly people, rural areas, and poverty-stricken regions. Specifically, the incidence in men was always higher than in women [1]. However, there are currently no prevention and control measures specifically targeted at the male population. Previous studies have shown that more frequent social communication and smoking could increase the risk of TB, especially sputum smear-positive TB [11-13]. Notably, compared with children under 15 years of age, the incidence in middle-aged and elderly people was high. Elderly people with low immune function may be particularly vulnerable to mycobacterial infections [14], and their treatment adherence is generally poor; therefore, the rate of unfavorable outcomes is higher in this sector of the population [14,15]. Moreover, up to 75.8% of residents aged ≥ 60 years in China have at least one chronic disease [16], making them more prone to complications and death when infected with TB [17]. Based on the above, the older TB cases should be proactively detected. Indeed, active TB detection and intervention in the older population may prove an effective public health strategy for reducing TB incidence [18].

The incidence of PTB in rural regions was significantly higher than in urban areas, and this result was similar to those from other regions in China. Cluster analysis also showed that regions in Henan province with high TB incidence were poverty-stricken counties (with a gross domestic product of <385.9 USD/person). In addition, our study also shows that bacteriological-positive PTB cases are concentrated in poverty-stricken counties, and the occurrence of bacteriological-positive PTB is associated with rural living. TB is associated with economic income [19], health and medical coverage, and culture and management. Regarding these aspects, rural regions lag behind urban areas. Therefore, these areas need to be designated as high-risk areas, or key areas where poverty alleviation should be strengthened, resident income should be increased, and medical service conditions should be improved.

In this study, we also observed that the proportion of bacteriological-positive PTB cases has decreased gradually. However, considering that bacteriological-positive cases are the main source of TB infection and that a single untreated patient with bacteriological-positive infection could infect 10-15 persons per year, new measures must be taken to achieve a significant reduction. At present, Henan province has carried out active surveillance on the fixed populations of rural areas to detect and treat all bacteriological-positive PTB patients, and also to design active detection strategies for key areas and key populations combined with detailed epidemiological analysis results.

The proportion of MDR-TB was high in China [9,20]; however, the dynamic compartmental model analysis revealed that improving the detection rate of, and increasing treatment success for, MDR-TB could yield the greatest reduction in the percentage of MDR-TB in all patients and new cases from 2015 to 2025. Based on this, 60% of smear-positive TB cases underwent tests for MDR-tuberculosis in Henan, including a routine drug sensitivity test and a rapid molecular drug sensitivity test, and second-line drugs, such as clofazimine and cycloserine, were provided to MDR-tuberculosis patients. However, the cure rates of clofazimine and cycloserine-containing regimens were only 68.7% and 66.0%, respectively [21]. Even if the cure rate of drugs against MDR-TB reaches 85.0% in the future, which could bring a greater reduction in the proportion of MDR-TB in all patients and new cases between 2015 and 2025, the WHO’s goal would still not be reached. Nevertheless, improving cure rates of DS-TB and MDR-TB remains the keystone to reducing incidence and mortality.

### Limitations

This study has some limitations. First, all the data used in this study were obtained from the TBIMS. However, drug resistance was not fully analyzed because the system contained incomplete drug resistance data. Second, no active detection and intervention data were obtained from the TBIMS. Hence, we only determined whether the WHO’s goal could be achieved through passive strategies.

### Conclusions

In China, from 1990 to 2020, 3 nationwide random surveys on TB were carried out to evaluate the effectiveness of the DOTS strategy. However, based on the internet-based TBIMS, we can now monitor the changes of TB epidemiological characteristics in real time while saving money, manpower, and time in the evaluation of the effect of different strategies. The local government has embarked on a series of nonfragmented TB-related intervention combinations, and the incidence of PTB and bacteriological-positive PTB has continued to decrease during the last 14 years; however, the WHO’s 2025 goal will not be reached. New active—rather than passive—strategies for detection and intervention should be formulated based on epidemiological characteristics while improving the cure rates of DS-TB and MDR-TB.

## Appendix

Multimedia Appendix 1 Detailed description of the dynamic compartmental model.

## Abbreviations

AOR: adjusted odds ratio
DOTS: directly observed treatment short-course
DS-TB: drug-susceptible tuberculosis
MDR-TB: multidrug-resistant tuberculosis
OR: odds ratio
PTB: pulmonary tuberculosis
TB: tuberculosis
TBIMS: Tuberculosis Information Management System
WHO: World Health Organization
