# Supplementary material for: Assessment of Strategies and Epidemiological Characteristics of Tuberculosis in Henan Province, China: Observational Study
Source: JMIR Public Health Surveill. 2021 Jan 22;7(1):e24830. doi: 10.2196/24830 (PMC7864773; doi:10.2196/24830)
Supplement: Multimedia Appendix 1 [file publichealth_v7i1e24830_app1.doc]

**The dynamic compartmental model**

1. **Model structures**

The dynamic compartmental model^1,2^ of TB was built to estimate the burden of TB epidemics, which was composed by a series of differential equations. According to tuberculosis (TB) infection status, we classified population into different group, i.e., susceptible, latent infection, active disease, cure after treatment, and failure/default/relapse after treatment. Susceptible may subsequently become latent infection status through TB infection transmission. The latent infection was divided into latent fast group or latent slow group in the model. Then disease progressed from latent infection to active disease, including smear positive status and smear negative status. Individuals with active TB could be natural cure, die, or seek treatment. Patients for seeking medical care were treated by the Centers for Disease Control and Prevention (CDC) systems or the hospital systems. With anti-tuberculosis treatment, they could either be cured, relapse or failure after the treatment and individuals with failed treatment could seek treatment in CDC or hospitals again. The dynamic compartmental model could be constructed using the disease progression and treatment path (Figure S1).


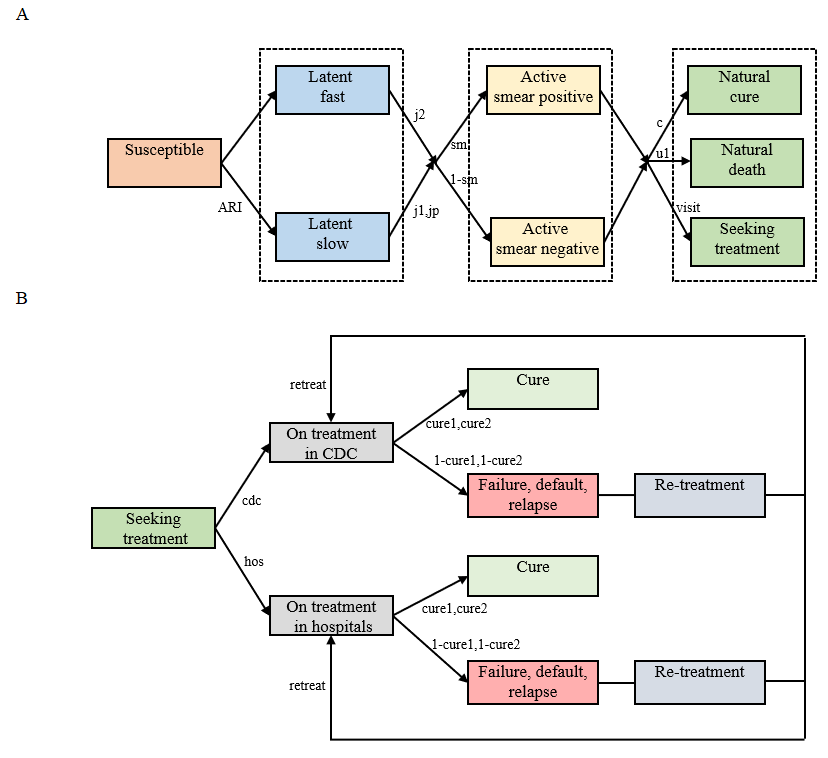


Figure S1 Disease progression and treatment path diagram. A. Disease schematic. B. Treatment pathway. The description of parameters showed in diagram was presented in Table S1.

1. **Model parameters**

In order to research whether Henan Province could achieve the post-2015 global targets, we fitted the burden of TB epidemics from 2005 to 2017 in Henan province based on a dynamic compartment model constructed by Lin et al^2^. As listed in the Table S1, thirty parameters including in the dynamic compartmental model could be divided into two categories. The first class included thirteen parameters that had unique characteristics of Henan Province. These parameters were determined by opinions of experts or data from Henan Province Bureau of Statistics. By asking the experts in Henan CDC or searching the data from Henan Province Bureau of Statistics, we set up the parameter values in line with Henan Province. The second class included thirteen TB natural history parameters that described disease progression of TB and four parameters that contained patient visit rate, patient visit rate of retreatment cases and probability of becoming MDR after treatment failure in CDC, or in hospitals. The settings of these four parameters were the same as those of the dynamic compartment model in China. Followed Lin et al^2^, we set up all seventeen parameters. Among them, the prior values of sixteen parameters were assumed log-normal with means and 95% ranges, triangular with lower and upper bound, or uniform distribution. By building the dynamic compartment model, parameters’ posterior distributions were simulated and its medians and 95% credible intervals were presented in Table S1.

Table S1 Input parameter values of the dynamic model

| **Notation** | **Parameter description** | **Prior values** | **Posterior values** | **Source** |
| --- | --- | --- | --- | --- |
| **First class parameters from unique characteristics of Henan Province** | | | | |
| u | Mortality rate | Time-varying | Same as prior value | Henan Province Bureau of Statistics |
| Birth | Birth rate | Time-varying | Same as prior value | Henan Province Bureau of Statistics |
| cdc | Patient visit rate (case detection rate) of new cases (year^-1^) | Time-varying | Time-varying | Expert opinion from CDC |
| hos | Proportion of detected cases treated in the hospital system | Time-varying | Time-varying | Expert opinion from CDC |
| c2h | Proportion of cases from the CDC system that received retreatment in the hospital system | 0.00 | 0.00 | Expert opinion from CDC |
| h2c | Proportion of cases from the CDC system that received retreatment in the hospital system | Time-varying | Time-varying | Expert opinion from CDC |
| DIAG_sp,cdc_ | Sensitivity of diagnostic algorithm smear-positive, CDC | 1.00 | Same as prior value | Expert opinion from CDC |
| DIAG_sn,cdc_ | Sensitivity of diagnostic algorithm smear-negative, CDC | 0.90 | Same as prior value | Expert opinion from CDC |
| DIAG_sp,hos_ | Sensitivity of diagnostic algorithm for smear-positive, hospital | 1.00 | Same as prior value | Expert opinion from CDC |
| DIAG_sn,hos_ | Sensitivity of diagnostic algorithm for smear-negative, hospital | 0.90 | Same as prior value | Expert opinion from CDC |
| dst | Probability of receiving drug susceptibility testing | 0.90 | Same as prior value | Expert opinion from CDC |
| cure1, cure2  cure2_secondline | Long-term cure rate of new (cure1) and retreatment (cure2) cases using first line drug and long-term cure rate of retreatment cases using second line drug (cure2_secondline) | See intervention strategies | Same as prior value | Expert opinion from CDC |
| sm | Percent smear positive among  incident cases of pulmonary TB | Log-normal:  0.40 (0.20, 0.60) | 0.40 (0.35, 0.50) | Expert opinion from CDC |
| **Second class parameters from Lin et al^2^** | | | | |
| b | Transmission parameter, smear positive (the number of people that one smear positive TB case can infect in a year in a completely susceptible population) | Log-normal:  6.50 (4.66, 9.07) | 6.04 (5.46, 6.70) | Lin et al^2^ |
| fitness | Relative fitness (that decreases the probability of transmission upon contact) of DR strain compared to DS strain | Uniform [0.60, 1.40] | 0.98 (0.83-1.12) | Lin et al^2^ |
| f_inf | Relative magnitude of transmission parameter, smear negative (relative to smear positive TB) | Log-normal:  0.22 (0.16, 0.30) | 0.22 (0.19, 0.24) | Lin et al^2^ |
| fail_inf | Relative magnitude of transmission parameter, failed cases (relative to new cases) | Triangular [0.80, 1.00] | 0.90 (0.87-0.93) | Lin et al^2^ |
| k | Transition rate from fast latent  period to slow latent period (year^-1^) | 1/5=0.2 | Same as prior value | Lin et al^2^ |
| j1 | Primary progression rate in the fast-latent period from new infection (year^-1^) | Log-normal:  0.030 (0.019, 0.047) | 0.027 (0.024-0.030) | Lin et al^2^ |
| j2 | Slow reactivation rate (year^-1^) | Log-normal:  0.00083(0.00059, 0.00119) | 0.0008 (0.0007, 0.00087) | Lin et al^2^ |
| im | Partial immunity that decreases probability of fast progression after re-infection | Log-normal:  0.79 (0.66, 0.88) | 0.79 (0.75, 0.83) | Lin et al^2^ |
| jp | Primary progression rate in the fast-latent period from re-infection (year^-1^) | = j1*(1-im) | 0.005 (0.0046-0.0068) | Lin et al^2^ |
| c | Natural cure rate for untreated  patients (year^-1^) | Log-normal:  0.100 (0.047, 0.210) | 0.10 (0.08, 0.13) | Lin et al^2^ |
| u1_sp_  u1_fail_sp_ | Death rate of those with active TB disease, smear positive cases (new and failed cases) (year^-1^) | Log-normal:  0.150 (0.081, 0.280)  0.050 (0.028, 0.090) | 0.16 (0.13, 0.19)  0.05 (0.04, 0.06) | Lin et al^2^ |
| u1_sn_  u1_fail_sn_ | Death rate of those with active TB disease, smear negative cases (new and failed cases) (year^-1^) | Log-normal:  0.050 (0.028, 0.090)  0.025 (0.020, 0.032) | 0.05 (0.04, 0.06)  0.025 (0.024, 0.027) | Lin et al^2^ |
| cv | Transition rate from smear-negative to smear positive TB (year^-1^) | Log-normal:  0.015 (0.010, 0.022) | 0.015 (0.013, 0.017) | Lin et al^2^ |
| visit | Patient visit rate (case detection rate) of new cases (year^-1^) | Log-normal:  1.00 (0.62, 1.60) | 0.99 (0.85, 1.16) | Lin et al^2^ |
| retreat_cdc_  retreat_hos_ | Patient visit rate of retreatment  cases (year^-1^) | CDC: 4.00 (3.05, 5.25)  Hospital: 1.00 (0.62, 1.60) | CDC: 4.00 (3.64, 4.39)  Hospital:0.99 (0.85, 1.19) | Lin et al^2^ |
| raf_cdc | Probability of becoming MDR after treatment failure, cdc | Log-normal:  0.05 (0.03, 0.09) | 0.05 (0.04, 0.06) | Lin et al^2^ |
| raf_hos | Probability of becoming MDR after treatment failure, hospital | Log-normal:  0.05 (0.03, 0.09) | 0.05 (0.04, 0.06) | Lin et al^2^ |

1. **Calibration**

We fitted the burden of TB epidemics from 2005 to 2017 in Henan province including incidence, mortality, the percentage of MDR in retreatment patients and all patients. The data were collected from epidemiological surveys and TB surveillance analysis reports. The sources of observed data were shown in Table S2.

Table S2 List of observed data

| Index | Calibration Data | Year and Value | Source |
| --- | --- | --- | --- |
| 1 | Incidence (per 100000) | 2005-2017 | CDC |
| 2 | Mortality (per 100000) | 2004: 6.57  2005: 6.58  2006: 5.18  2007: 4.83  2008: 3.90  2010: 3.30 | Hu et al^3^,  Murray et al^4^ |
| 3 | Percent of MDR in all patients | 2001: 12.9  2007: 6.80  2010: 6.67  2014: 6.17  2015: 6.42  2016: 6.28  2018: 5.68 | Zheng and Zhen^5^ |
| 4 | Percent of MDR in new patients | 2001: 7.8  2007: 3.0  2014: 4.71  2015: 4.69  2016: 4.87  2018: 3.63 | Zheng and Zhen^5^ |
| 5 | Percent of MDR in retreatment patients | 2001: 36.6  2007: 21.7  2014: 17.04  2015: 18.06  2016: 16.15  2018: 15.03 | Zheng and Zhen^5^ |

Adopting the Bayesian melding approach^6^, we calibrated the model to represent the burden of TB in the Henan province. First, 50,000 simulations were performed and for each simulation, model parameters (Table S1) were selected randomly from prior distributions of these parameters. Second, for each simulation, a likelihood score could be computed by comparing observed data with fitted values. Third, we resampled 50,000 times based on likelihood score and got the posterior distributions of model parameters. Details about the calibration process referred to Lin et al^2^. Results of model calibration were showed in Figure S2.


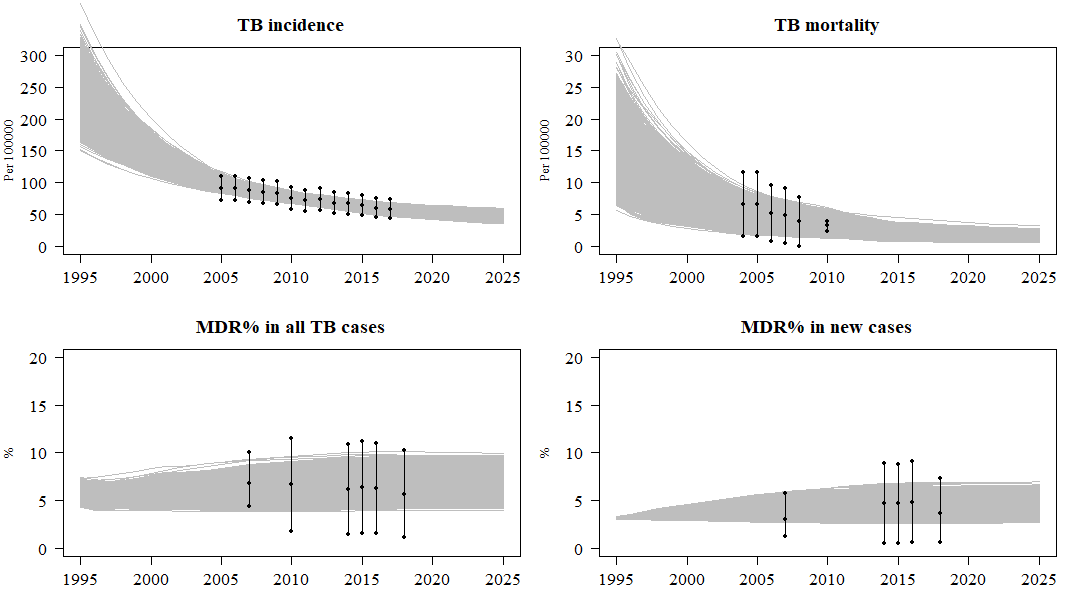


Figure S2 Results of model calibration. The black dots and bars represent the observed data and its 95% confidence intervals. The grey lines represent 50,000 posterior simulations.

1. **Future intervention strategies**

In order to describe the potential impact of current and alternative control measures on the epidemiology of TB in Henan province, we simulated four scenarios and investigated the spread of disease.

**Scenario 1: Current Status in Henan**

A total of 90% of people who suffer from PTB are treated at the Henan CDC system, and 10% of the patients tend to choose the hospital system to cure TB. In the CDC system, long-term cure rate is 92% and 87% for new cases and retreatment cases, respectively, and 35% for multidrug-resistant tuberculosis (MDR-TB) using first-line anti-TB drugs. In the hospital system, the long-term cure rate is 80% and 75% for new cases and retreatment cases, respectively, and 30% for MDR-TB using first-line drugs. 60% smear-positive patients receive testing for drug resistance and patients detected with MDR tuberculosis are placed on second-line therapy. The long-term cure rate for MDR tuberculosis with second-line anti-TB therapy is 50%.

**Scenario 2: Improving treatment outcome for DS-tuberculosis**

Using new and better treatment regimen for DS-tuberculosis, the long-term cure rates in CDC or hospital systems are both 92% for new treatment and 90% for retreatment.

**Scenario 3: Increasing treatment success for MDR tuberculosis**

New treatment regimen for MDR tuberculosis are widely and effectively adopted. 90% of TB smear-positive patients receive resistance testing, and the long-term cure rate with second-line drugs for MDR-TB is 82%.

**Scenario 4: Combined TB control programme**

In scenario 4, it represents a combined TB control programme in which scenarios 1，2 and 3 are delivered simultaneously. The treatment success rate for DS-TB increases using a bunch of measures and the detection and treatment of MDR tuberculosis are improved.

**References**

1. Menzies NA, Cohen T, Lin HH, et al. Population health impact and cost-effectiveness of tuberculosis diagnosis with Xpert MTB/RIF: a dynamic simulation and economic evaluation. *PLoS Med* 2012; **9**(11): e1001347.

2. Lin HH, Wang L, Zhang H, et al. Tuberculosis control in China: use of modelling to develop targets and policies. *Bull World Health Organ* 2015; **93**(11): 790-798.

3. Hu J, Wang LX, Chen W, et al. Analysis on status and trend of tuberculosis mortality from 2004 to 2008 in China. *Chinese Journal of Antituberculosis* 2011; **33**(4): 232-237.

4. Murray CJ, Ortblad KF, Guinovart C, et al. Global, regional, and national incidence and mortality for HIV, tuberculosis and malaria during 1990-2013: a systemic analysis for the Global Burden of Disease Study 2013. *Lancet* 2014; **384**(9947): 1005-1070.

5. Zheng CF, Zhen XA. Analysis of multi-drug resistance status of tuberculosis bacteria in Henan Province. *Henan Journal of Preventive Medicine* 2017; **28**(7): 514-515.

6. Alkema L, Raftery AE, Brown T. Bayesian melding for estimating uncertainty in national HIV prevalence estimates. *Sex Transm Infect* 2008; **84 Suppl 1**: i11-i16.
